# Intestinal Epithelial Cells Regulate Gut Eotaxin Responses and Severity of Allergy

**DOI:** 10.3389/fimmu.2018.01692

**Published:** 2018-08-03

**Authors:** Eunsoo Kim, Melanie Lembert, Ghaith M. Fallata, John C. Rowe, Tara L. Martin, Abhay R. Satoskar, Nicholas V. Reo, Oleg Paliy, Estelle Cormet-Boyaka, Prosper N. Boyaka

**Affiliations:** ^1^Department of Veterinary Biosciences, The Ohio State University, Columbus, OH, United States; ^2^Department of Biochemistry and Molecular Biology, Boonshoft School of Medicine, Wright State University, Dayton, OH, United States; ^3^Department of Pathology, The Ohio State University, Columbus, OH, United States

**Keywords:** intestinal epithelial cells, allergy, eotaxins, NF-κB, eosinophils

## Abstract

Intestinal epithelial cells (IECs) are known to regulate allergic sensitization. We addressed the role of the intrinsic IKKβ signaling in IECs in the effector phase of allergy following oral allergen challenge and its impact on the severity of responses is poorly. Upon orally sensitization by co-administration of ovalbumin with cholera toxin as adjuvant, wild-type and mice lacking IKKβ in IECs (IKKβ^ΔIEC^ mice) developed similar levels of serum IgE and allergen-specific secretory IgA in the gut. However, subsequent allergen challenges in the gut promoted allergic lower responses in KKβ^ΔIEC^ mice. Analysis of cytokines and chemokines in serum and gut tissues after oral allergen challenge revealed impaired eotaxin responses in IKKβ^ΔIEC^ mice, which correlated with lower frequencies of eosinophils in the gut lamina propria. We also determined that IECs were a major source of eotaxin and that impaired eotaxin production was due to the lack of IKKβ signaling in IECs. Oral administration of CCL11 to IKKβ^ΔIEC^ mice during oral allergen challenge enhanced allergic responses to levels in wild-type mice, confirming the role of IEC-derived eotaxin as regulator of the effector phase of allergy following allergen challenge. Our results identified targeting IEC-derived eotaxin as potential strategy to limit the severity of allergic responses to food antigens.

## Introduction

Food allergy is a hypersensitive reaction against harmless food materials such as cow’s milk, hen’s egg, or peanut, to name a few (1, 2). Food allergy can be acquired after loss of immunological tolerance (mucosal tolerance) to food antigens. Because of shared moieties, sensitization to food allergens can also lead to allergies to environmental, plant, or other unrelated products (3). According to the CDC report, young students who have food allergy also have higher incidence of other allergies such as asthma or dermatitis (4). Incidence of allergies is growing (2, 3, 5) and environmental factors, including pollutants, drugs, antibiotics, and stress are believed to contribute to this trend.

Allergic responses occur after a cascade of events that can be divided into a sensitization or priming phase, which is when the host develops adaptive immune responses against the allergens, and an effector phase. The effector phase generates the clinical signs of allergy and is triggered by the re-exposure of cells and molecules of the adaptive immunity to the allergens. Allergic symptoms are different according to the sites where allergic responses occur, but most of the food allergic responses are associated with IgE, mast cell degranulation, and eosinophilia (6). Intestinal epithelial cells (IECs) are the first cells encountered by ingested allergens. The role of these cells in allergy was initially believed to be restricted to the regulation of intestinal permeability to luminal content. It is now well established that IECs produce cytokines, including IL-25, IL-33, and thymic stromal lymphopoietin (TSLP), which help the development of Th2 cells necessary for production of IgE and allergic responses (7–10). The nuclear factor-κB (NF-κB) pathway was shown to play a major role in cytokine production by IECs. In this regard, NF-κB is a transcriptional factor that regulates immunological response including inflammation and lymphoid organogenesis (11). Signals through the canonical NF-κB pathways are regulated by the inhibitor-κB kinase (IKKβ) (12). We have previously shown that cell-specific ablation of IKKβ in IECs (IKKβ^ΔIEC^ mice) alters the profile of allergen-specific T helper cells responses (13). These studies also showed that orally sensitized IKKβ^ΔIEC^ mice develop lower levels of allergic lung inflammation than their wild-type counterpart after nasal allergen challenge (13). The protective effect observed in IKKβ^ΔIEC^ mice correlated with higher levels of allergen-specific IgA in the lungs and bronchoalveolar lavages suggesting that allergen-specific IgA present in the airways limited the extent of allergic airway inflammation in these mice (13). However, it remains unclear whether allergen-specific secretory IgA (SIgA) present in the intestinal lumen and in gut-associated lymphoid tissues could also prevent or limit the severity of allergic inflammation in the gastrointestinal (GI) tract. Also unclear is how the lack of IKKβ in IECs impacts the effector phase of allergic responses after oral allergen challenge and the development of clinical signs of allergy.

Using the well-established model of oral sensitization in the presence of cholera toxin (CT) as adjuvant (13–15), we addressed the role of IECs during the effector phase of allergy response subsequent to oral allergen challenge. Our data show that lack of IKKβ in IECs prevents the development of allergic responses and identifies the NF-κB eotaxin axis as a mechanism through which IECs regulate the severity of allergic responses to ingested allergens.

## Results

### Lack of Intestinal Epithelial Cell-Intrinsic IKKβ Lowers Allergic Responses to Ingested Antigens

**Figure 1:**
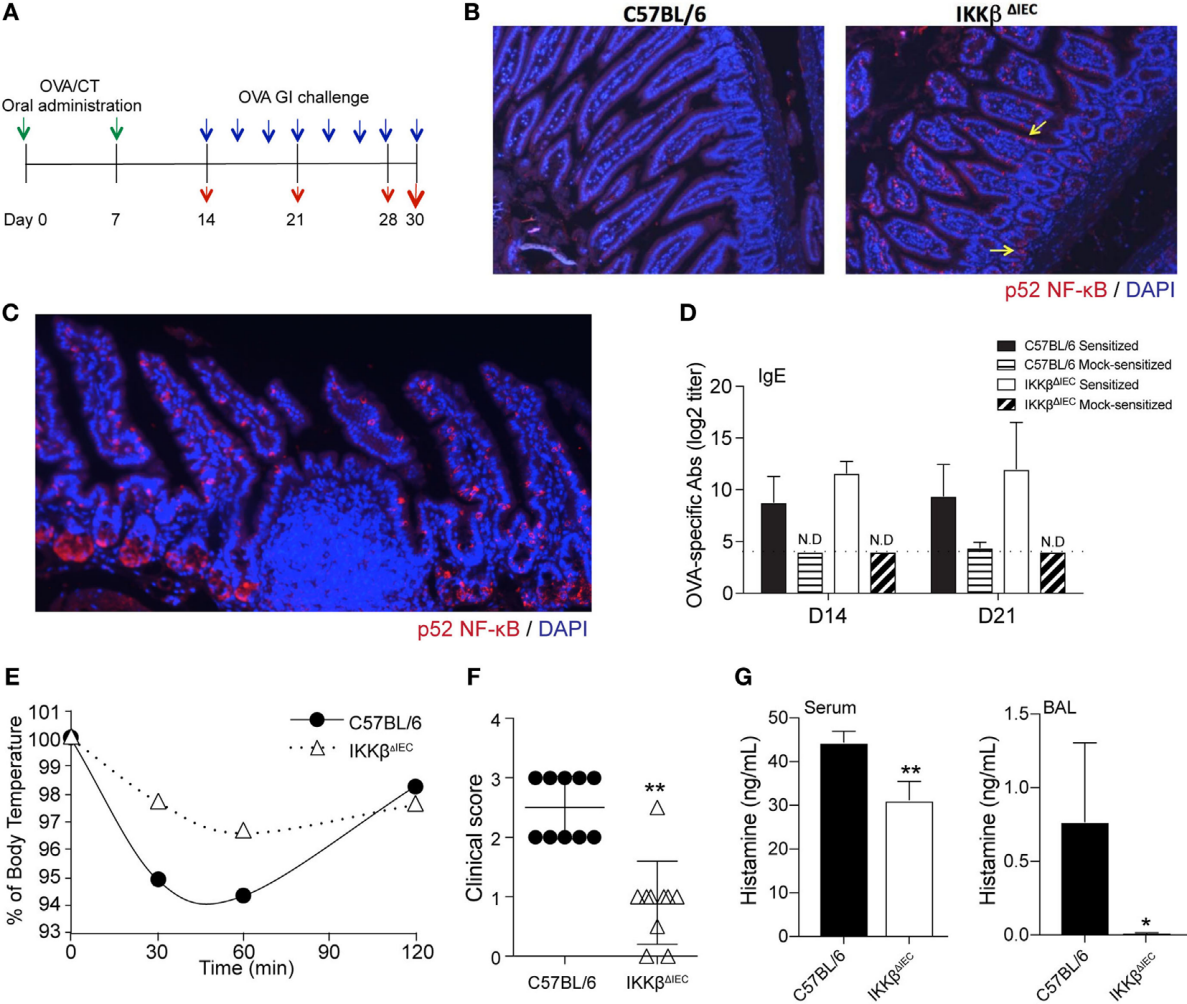
Mice with selective IKKβ-deficiency in intestinal epithelial cells have lower clinical signs of allergy after oral allergen challenge. **(A)** Experimental scheme. Wild-type C57BL/6 and IKKβ^ΔIEC^ mice were orally sensitized by ovalbumin (OVA) allergen and cholera toxin (CT) as adjuvant (green arrows), and then orally challenged with OVA (blue arrows). Serum or lung tissues were collected at days 14, 21, 28, and 30 to analyze antibodies responses or clinical signs of allergy (red arrows). **(B)** Expression of the non-canonical NF-κB pathways in the intestine of naïve wild-type C57BL/6 and IKKβ^ΔIEC^ mice and **(C)** expression of the non-canonical NF-κB pathways in intestinal villi, crypts, and Peyer’s patches of IKKβ^ΔIEC^ mice. Sections of small intestinal tissues were stained with an anti-p52 NF-κB antibody and counterstained with DAPI to visualize the nuclei. **(D)** Allergen-specific serum IgE. Mice were sensitized by oral administration of OVA (1 mg) and CT (15 µg). Serum samples were collected on days 14 and 21 and OVA-specific IgE titers were determined by enzyme-linked immunosorbent assay (ELISA). The results are expressed as mean log_2_ titers ± 1 SD and are from at least five experiments and five mice/group. **(E)** Variation (%) of surface body temperature after oral challenge with OVA (50 mg) on day 30. **(F)** Clinical scores. Mice were orally challenged with OVA (50 mg) and clinical scores recorded according to the behavior criteria described in Section “Materials and Methods.” **(G)** Histamine levels after nasal allergen challenge with OVA on day 30. Serum and BAL samples were collected 2 h after allergen challenge and histamine levels measured by ELISA. Data are expressed as mean ± SD. *p < 0.05; **p < 0.01.

To address the role of intestinal epithelial cell IKKβ in allergic responses in the gut, age-matched wild-type C57BL/6 and IKKβ^ΔIEC^ mice were co-housed and orally sensitized by administration of ovalbumin (OVA) and CT as adjuvant, and then orally challenged with OVA alone (Figure 1A). Since IKKβ^ΔIEC^ mice are defective in canonical NF-κB pathway signaling in IECs, we first examined whether a compensatory pathway occurred in the gut of these mice. Analysis of gut tissues revealed activation of the non-canonical NF-κB pathway in intestinal villi and crypts of IKKβ^ΔIEC^ mice that was not observed in the wild-type counterpart (Figure 1B). It is worth noting that p52 NF-κB was not detected in the Peyer’s patches (Figure 1C), which suggests that activation of non-canonical NF-κB in IKKβ^ΔIEC^ mice is restricted to epithelial cells. The lack of canonical NF-κB signaling and subsequent activation of the non-canonical NF-κB in IKKβ^ΔIEC^ mice did not affect allergic sensitization since wild-type and IKKβ^ΔIEC^ mice developed similar levels of allergen-specific IgE responses following oral sensitization (Figure 1D).

Oral allergen challenge of sensitized mice induced a significant drop of body temperature in wild-type mice (Figure 1E), which was consistent with a significant change in behavior. Thus, these mice showed signs of allergy (i.e., low activity, scratching of nose and mouth, and self-isolation) with an average clinical score of 2.5 (Figure 1F) and high histamine levels in the serum (Figure 1G). Consistent with the fact that allergic reactions can occur at distant mucosal sites following ingestion of food antigen, high levels of histamine were also detected in the BAL fluids (Figure 1G). Despite containing similar levels of allergen-specific serum IgE to control wild-type mice, the IKKβ^ΔIEC^ mice exhibited a significantly lower drop in body temperature following oral allergen challenge, which was in line with their significantly lower clinical signs of allergy and histamine levels (Figures 1E–G).

### Allergen-Specific SIgA Unlikely Reduces the Severity of Allergy in IKKβ^ΔIEC^ Mice Following Oral Allergen Challenge

**Figure 2:**
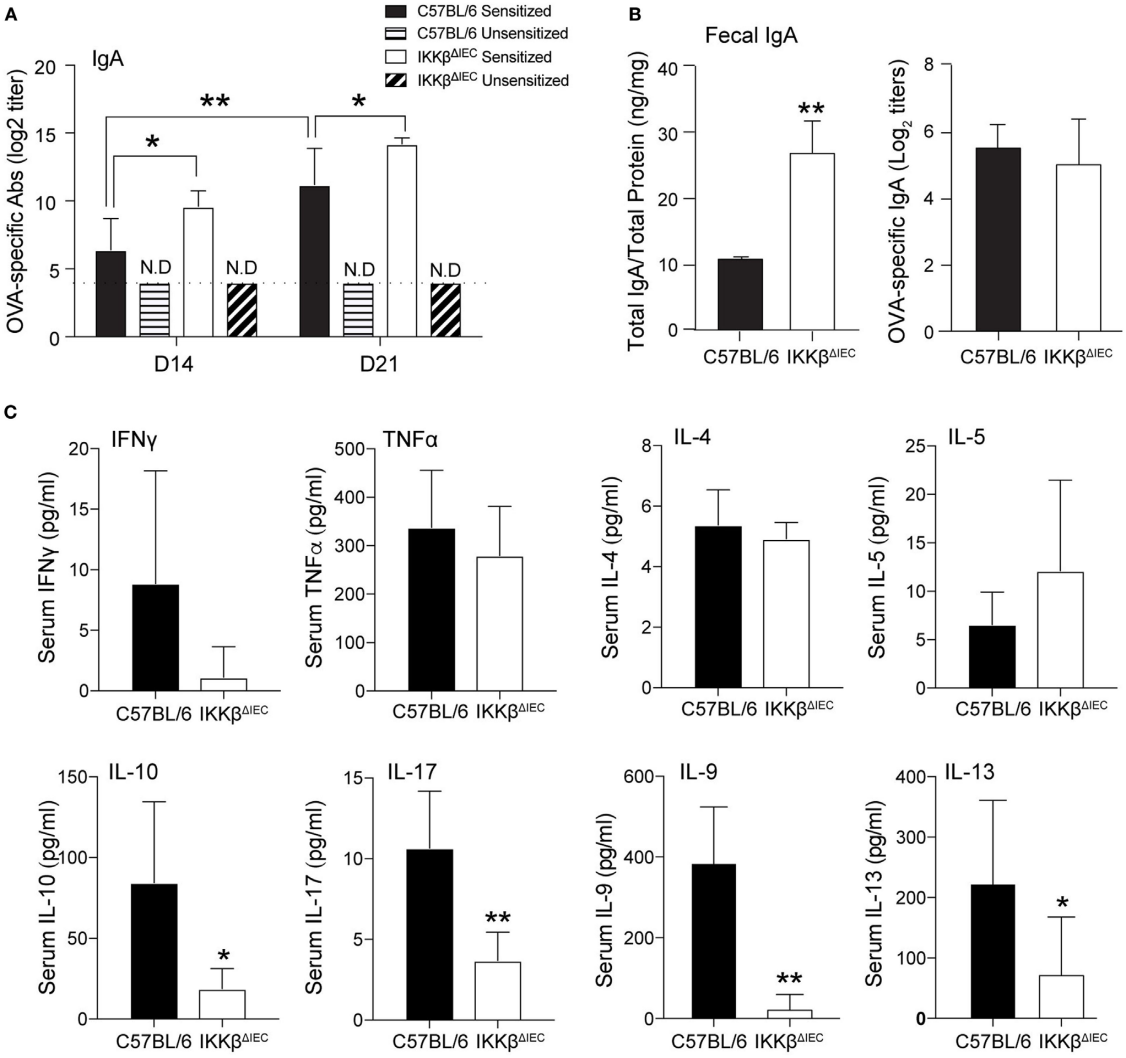
Allergen-specific secretory IgA (SIgA) and serum cytokine responses of IKKβ^ΔIEC^ mice after oral sensitization and oral allergen challenge. **(A)** Allergen-specific serum IgA. **(B)** Total and allergen-specific SIgA in fecal extracts. Mice were sensitized by oral administration of ovalbumin (OVA) (1 mg) and cholera toxin (15 µg). Serum were collected on days 14 and 21, and fecal samples were collected on day 30. Total and OVA-specific IgA were assessed by enzyme-linked immunosorbent assay. **(C)** Serum cytokines. Serum samples were collected 2 h after allergen challenge on day 30, and the concentrations of cytokines were evaluated by a multiplex assay. Data are expressed as mean ± SD. *p < 0.05; **p < 0.01 and are from at least five experiments and five mice/group.

Secretory IgA are believed to limit the entry of pathogens and allergens into the host (16). Accordingly, the presence of allergen-specific IgA in the BAL was shown to correlate with lower levels of allergic inflammation in IKKβ^ΔIEC^ mice after nasal allergen challenge (13). As previously reported, oral sensitization induced higher levels of allergen-specific serum IgA in IKKβ^ΔIEC^ mice (Figure 2A). The total amounts of IgA measured in fecal extracts were significantly higher in IKKβ^ΔIEC^ mice at days 14 and 21 (Figure 2B). Surprisingly, no difference was noted between OVA-specific SIgA responses in fecal extract of IKKβ^ΔIEC^ and control wild-type mice.

Since SIgA levels failed to provide clues on how IKKβ^ΔIEC^ mice only developed minimal signs of allergic responses following oral allergen challenge, we analyzed cytokines present in the serum of these mice. No significant difference was noted between the levels of Th1 cytokines (i.e., IFNγ, TNFα) in the serum of control wild-type and IKKβ^ΔIEC^ mice following oral allergen challenge (Figure 2C). Interestingly, serum Th2 cytokines were differently affected by the absence of the canonical NF-κB pathway in IECs. Thus, while wild-type and IKKβ^ΔIEC^ mice expressed similar levels of serum IL-4 and IL-5, IKKβ^ΔIEC^ mice exhibited reduced levels of IL-9 and IL-13 (Figure 2C). Serum IL-10 and IL-17 levels were also lower in IKKβ^ΔIEC^ mice (Figure 2C).

### IKKβ Mice Have Fewer Eosinophils in the Gut

**Figure 3:**
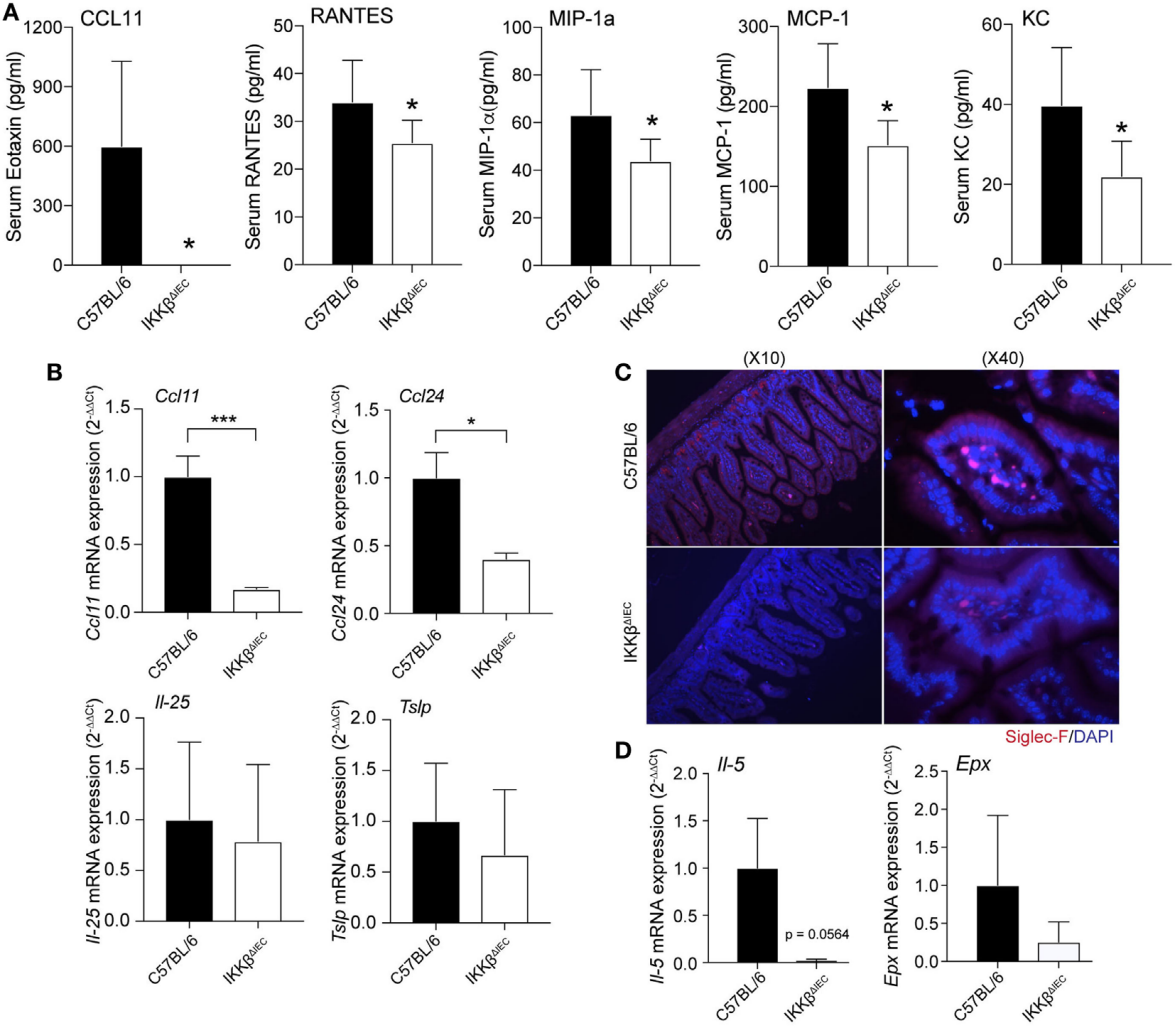
Serum chemokines and gut eosinophil levels in IKKβ^ΔIEC^ mice following oral sensitization and oral allergen challenge. **(A)** Serum chemokines. Serum samples were collected 2 h after allergen challenge on day 30 and the concentrations of chemokines were evaluated by multiplex assay. Data are expressed as mean ± SD. *p < 0.05; **p < 0.01 and are from at least five experiments and five mice/group. **(B–D)** Analysis of small intestinal tissues collected on day 30 and 2 h after allergen challenge of wild-type and IKKβ^ΔIEC^ mice. **(B)** Eotaxin-1 (*Ccl11)*, eotaxin-3 *(Ccl24*), *il-25*, and *Tslp* mRNA levels. **(C)** Eosinophils in small intestine. Thin sections of small intestinal tissues were stained with an anti-Siglec-F antibody and counterstained with DAPI to visualize nuclei. **(D)** *Il-5* and eosinophil peroxide (*Epx*) mRNA levels. The mRNA data are expressed as mean ± SD. *p < 0.05; **p < 0.01, and are from at least five experiments and five mice/group.

Development of allergic responses requires induction of allergen-specific IgE and subsequent activation of mast cells, basophils, and eosinophils (17). Analysis of serum chemokines after oral allergen challenge revealed that the levels of eotaxin-1 (or CCL11), a chemokine that selectively recruits eosinophils, were significantly reduced in IKKβ^ΔIEC^ mice (Figure 3A). These mice also showed significantly lower levels of inflammatory chemokines (e.g., RANTES, MIP-1a, MCP-1, and KC) (Figure 3A). To further establish that the lower serum CCL11 levels were an indication of impaired eosinophil recruitment in IKKβ^ΔIEC^ mice, we analyzed expression of transcripts for eotaxins (*Ccl11* and *Ccl24*) and other Th2-promoting cytokines (*Il-25* and *Tslp*) in small intestinal tissues collected after the allergen challenges. Wild-type and IKKβ^ΔIEC^ mice showed no difference in the levels of *Il-25* and *Tslp* mRNA. On the other hand, the mRNA levels of the eosinophil chemoattractant *Ccl11* and *Ccl24* were lower in the small intestine of IKKβ^ΔIEC^ than in wild-type mice (Figure 3B). Histological analysis of small intestinal sections stained with an anti-Siglec-F antibody showed the presence of eosinophils in the gut of control wild-type and IKKβ^ΔIEC^ mice, and there was no significant difference in the number of eosinophils in these mice at the steady state (not shown). Conversely, oral allergen challenge increased the number of eosinophils in the small intestine of wild-type mice, but not in IKKβ^ΔIEC^ mice (Figure 3C). Consistent with histology, mRNAs levels of eosinophil growth factor (*Il-5*), eosinophil peroxidase (*Epx*) were both lower in small intestinal tissues of IKKβ^ΔIEC^ than wild-type mice following oral allergen challenge (Figure 3D).

### Intrinsic Signals in Epithelial Cells Impair Eotaxin Responses in the Gut of IKKβ^ΔIEC^ Mice

**Figure 4:**
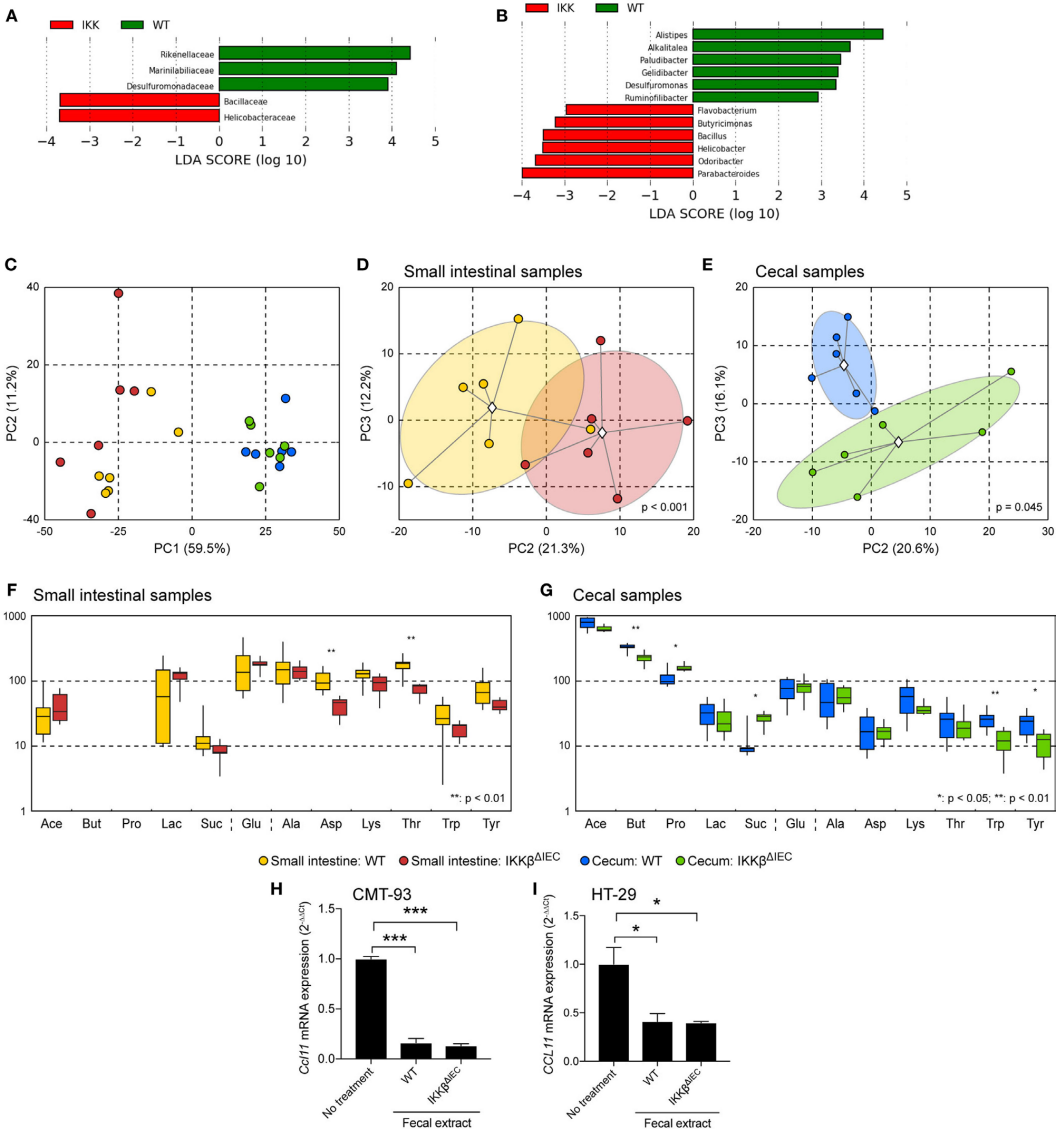
Commensal bacteria and metabolite profiles are altered in the gut of naïve IKKβ^ΔIEC^ mice. **(A,B)** Linear discriminant analysis (LDA) of the abundances of commensal gut bacteria. Freshly emitted fecal pellet were normalized by weight and microbial composition determined by 16S RNA analysis. **(A)** LDA scores at the family level. **(B)** LDA scores at the genus level. **(C–G)** Metabolite profiling. **(C–E)** Principal component analyses (PCA) of metabolite profiles. Different groups are denoted by colors as shown in the legend. Group clouds represent areas of three SEs around the group centroid (diamond). Percent of total variance captured by each principal component is shown in parentheses. *P* values indicate the statistical significance of group separation in the first three components of the PCA space as assessed by permutation analysis of Davies-Bouldin index. **(C)** Small and large intestines; **(D)** small intestine; **(E)** large intestine. **(F)** Distribution of concentrations of major metabolites in the small intestine. **(G)** Distribution of concentrations of major metabolites in the large intestine. Boxplot whiskers depict minimum and maximum values for each group. *P* values were calculated using two-tailed *T*-test, *p < 0.05; **p < 0.01 (five mice/group). Abbreviations: Ace, acetic acid; But, butyric acid; Pro, propionic acid; Lac, lactic acid; Suc, succinic acid; Glu, glucose; Ala, alanine; Asp, aspartic acid; Lys, lysine; Thr, threonine; Trp, tryptophan; Tyr, tyrosine. Note that there were no discernable nuclear magnetic resonance peaks for butyric and propionic acids in any small intestinal samples. **(H,I)** CCL11 mRNA expression of in murine CMT-93 **(H)** and human HT-29 intestinal epithelial cells **(I)**. Cells were cultured for 12 h in the presence of bacterial-free fecal extracts from wild-type and IKKβ^ΔIEC^ mice. *p < 0.05; **p < 0.01; ***p < 0.001 compared to no treatment controls.

The altered eotaxin responses in the gut of IKKβ^ΔIEC^ mice could result from endogenous factors due to lack of IKKβ signaling or exogenous factor *via* the gut microbiota. We have previously shown that IKKβ^ΔIEC^ mice display a gut microbiota dysbiosis that is further enhanced after oral administration of CT (13). Linear discriminant analysis (LDA) of commensal bacteria at the family (Figure 4A) and genus (Figure 4B) levels clearly identified bacteria associated with the presence of a functional or non-functional IKKβ in epithelial cells. Since the presence of butyrate-producing bacteria is often associated with protection against the development of allergic responses (18–21), we also analyzed the profile of metabolites present in the small and large intestines of wild-type and IKKβ^ΔIEC^ mice (Figures 4C–G). Asparagine and threonine levels were significantly reduced in the small intestine of IKKβ^ΔIEC^ mice. Further, the large intestine of these mice contained lower levels of butyrate, tryptophan, and tyrosine while propionate and succinate levels were increased. We also investigated whether these metabolites or other molecules in the fecal contents of wild-type and IKKβ^ΔIEC^ mice differentially affected eotaxin expression. We found that addition of bacteria-free fecal material extracts inhibits CCL11 mRNA expression by murine (CMT93) and human (HT-29) intestinal epithelial cell lines, regardless of the wild-type or IKKβ^ΔIEC^ mouse origin of the fecal materials (Figures 4H,I). Thus, neither the nature of the bacteria nor the metabolites present in the gut of IKKβ^ΔIEC^ mice could explain the altered CCL11 responses.

**Figure 5:**
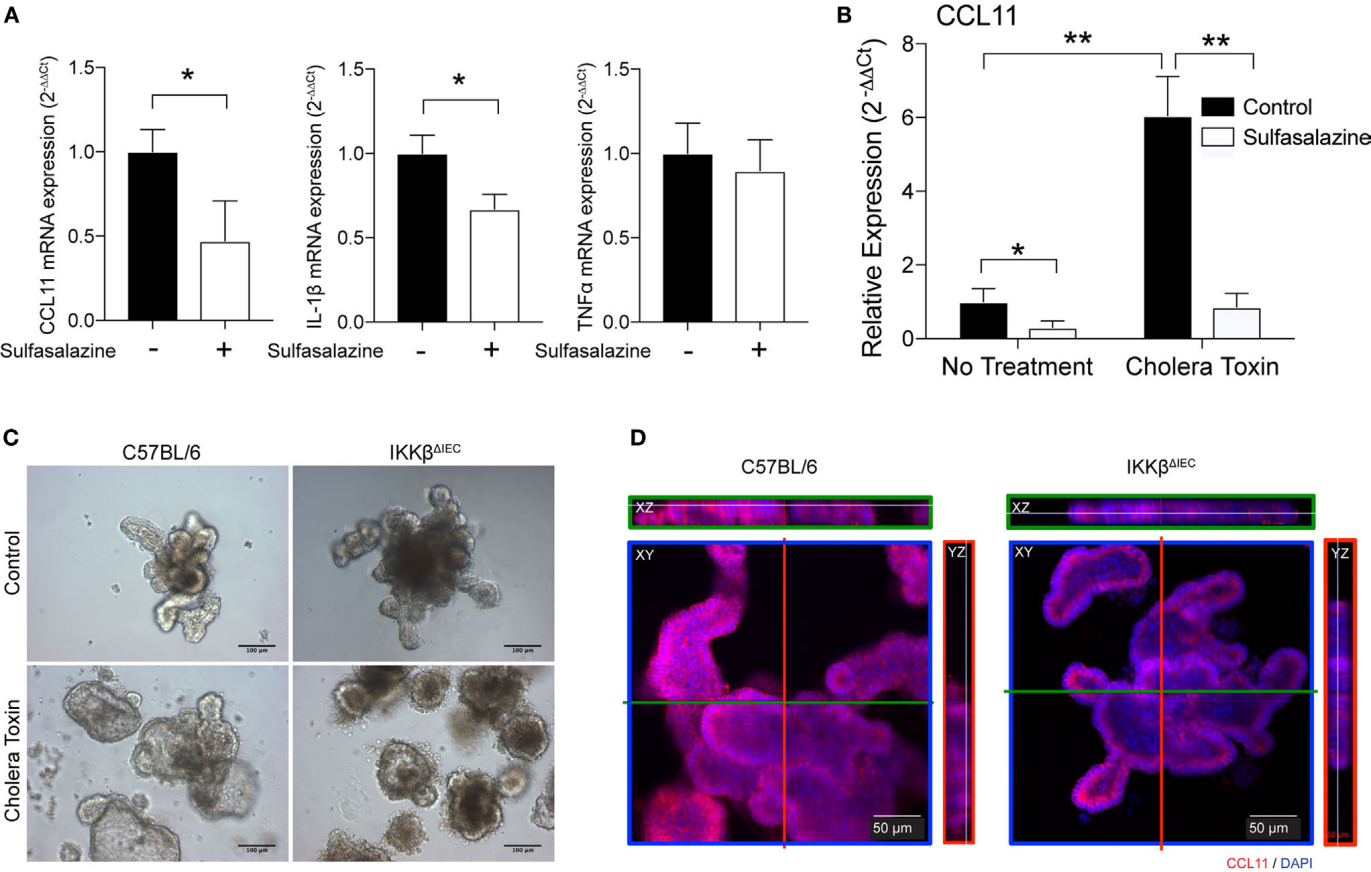
CLL11 expression in human epithelial cells and organoids of wild-type and IKKβ^ΔIEC^ mice. **(A,B)** The human HT-29 epithelial cells were cultured in the absence or presence of sulfasalazine, a specific pharmacological inhibitor (50 µM) of the canonical NF-κB pathway, without **(A)** or with **(B)** stimulation with cholera toxin (CT) (1 µg/ml). **(A)** Relative *CCL11, IL-1*β, and *TNF-*α mRNA were compared to control cells non-treated with sulfasalazine. (B) Relative *CCL11* mRNA levels in HT29 epithelial cells treated CT, in the absence or presence of sulfasalazine. The mRNA levels were measured by real-time RT-PCR 8 h after exposure to sulfasalazine. Data are expressed as mean ± SD. *p < 0.05; **p < 0.01, and are from four independent studies. **(C,D)** Organoids derived from wild-type and IKKβ^ΔIEC^ mice. **(C)** Organoids cultures were incubated for 16 h in the presence of CT (1 µg/ml). **(D)** Expression of CCL11 protein in organoids derived from wild-type and IKKβ^ΔIEC^ mice. Organoid cultures were stained with an anti-CCL11 antibody and counterstained with DAPI to visualize nuclei before analysis with a confocal microscope.

We next tested if IECs could represent a major source of eotaxin in the gut and whether inhibition or lack of IKKβ signaling could be sufficient to impair CCL11 production. For this purpose, we used human HT-29 epithelial cells and blocked NF-κB signaling by using sulfasalazine, a pharmacological inhibitor of the canonical NF-κB pathway. Addition of sulfasalazine to cultures of epithelial cells significantly reduced *CCL11* mRNA levels (Figure 5A). This treatment did not affect all cytokine mRNA responses since *IL-1*β, but not *TNF*α mRNA were downregulated (Figure 5A). We also found that HT-29 epithelial cells cultured in the presence of sulfasalazine have significantly lower *CCL11* mRNA responses to exposure to CT (Figure 5B). To further confirm that IECs were the source of impaired eotaxin responses in the gut of IKKβ^ΔIEC^ mice, we developed organoid cultures from intestinal crypt cells of wild-type and of IKKβ^ΔIEC^ mice (Figure 5C). As depicted in Figure 5C, cells from IKKβ^ΔIEC^ mice formed organoid that resembled those of control mice. Furthermore, these organoids were functional as they accumulated fluid after stimulation with CT (Figure 5C). It is worth noting that organoids of IKKβ^ΔIEC^ mice seemed less inflated after stimulation with CT than those of control wild-type mice, and this was consistent with the reduced intestinal fluid accumulation seen after these mice were given CT by oral gavage (not shown). Finally, immunofluorescence analysis of organoids stained with an anti-CCL11 antibody demonstrated that although organoids of IKKβ^ΔIEC^ mice expressed CCL11, their levels were significantly lower than those detected in organoids derived from control wild-type mice (Figure 5D).

### Oral Administration of CCL11 Protein Restores Clinical Signs of Allergy in IKKβ^ΔIEC^ Mice

**Figure 6:**
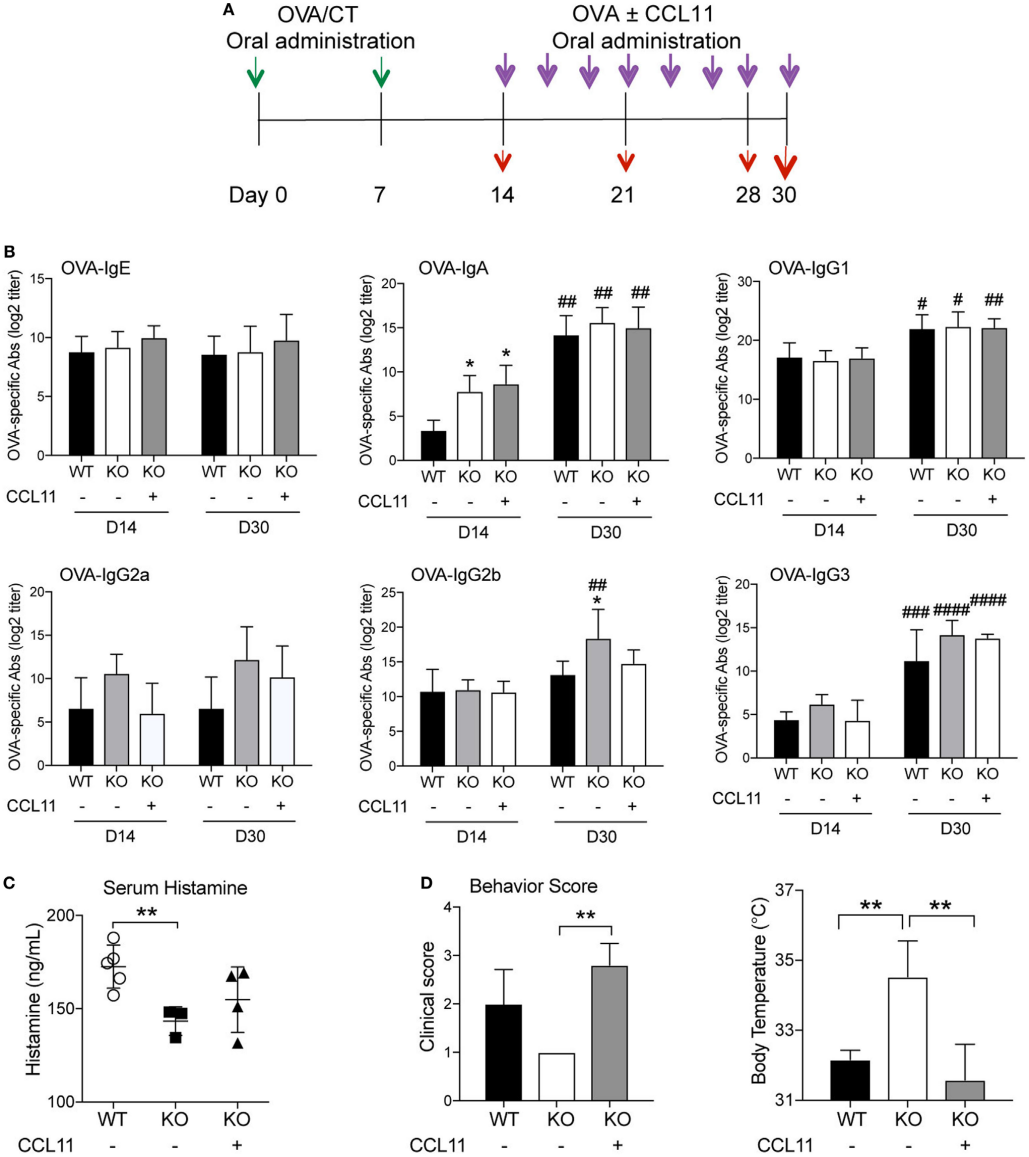
Oral CCL11 at the time of allergen challenge restores allergic responses in IKKβ^ΔIEC^ mice. **(A)** Experimental scheme. Oral sensitized IKKβ^ΔIEC^ mice with ovalbumin (OVA) and CT (green arrows) were orally administered OVA allergen with or without recombinant CCL11 protein (purple arrows). Serum or lung tissues were collected at days 14, 21, 28, and 30 to analyze antibodies responses or clinical signs of allergy (red arrows). **(B)** Allergen-specific serum Ab responses. Serum samples were collected on days 14 and 30 and OVA-specific Ab titers were determined by enzyme-linked immunosorbent assay. Data are expressed as mean ± SD. *p < 0.05; **p < 0.01 when compared to wild-type (WT). ^#^*p* < 0.05, ^##^*p* < 0.01; ^###^*p* < 0.001; ^####^*p* < 0.0001 compared today 14. **(C)** Serum histamine levels, **(D)** clinical scores, and surface body temperature after oral allergen challenge with OVA. Data are expressed as mean ± SD. *p < 0.05; **p < 0.01 (*n* = 5 mice/group).

Most studies addressed the regulatory effect of cytokines *in vivo* after parenteral injection. However, cytokines were also administered orally to test their role/effect in a variety of experimental systems (22–26). In our own previous studies, oral delivery of IL-12 failed to result in significant levels of serum IL-12 (27). However, this treatment was able to promote IgG2a/c and suppress IgE antibody responses in mice orally immunized with CT as adjuvant (27), indicating that cytokine can be administered orally to assess their effect in mucosal tissues and to a lesser extent in the bloodstream. Thus, to confirm the central role of intestinal epithelial cell-derived CCL11 in the effector phase of allergic response, orally sensitized IKKβ^ΔIEC^ mice were orally administered recombinant CCL11 protein at the time of oral allergen challenges (Figure 6A). Oral treatment with CCL11 did not induce major changes in OVA-specific antibody responses (Figure 6B). Thus, it only affected OVA-specific IgG2b, which were downregulated after ingestion of CCL11 recombinant protein and reached similar levels than in control wild-type mice (Figure 6B). Interestingly, allergic response to oral allergen challenge were restored in IKKβ^ΔIEC^ mice after treatment with CCL11. More specifically, IKKβ^ΔIEC^ mice that received CCL11 had serum histamine levels that were closer to those of control wild-type mice after oral allergen challenge (Figure 6C). Furthermore, other clinical signs of allergy including behavior score and body surface temperature were restored to levels similar to wild-type C57BL/6 after ingestion of CCL11 (Figure 6D).

## Discussion

Epithelial cells sense intestinal microbes and produce initial innate signals that regulate host response to microbes and food products. It is now well established that health is associated with balanced gut microbiome, while dysbiosis can lead to a variety of diseases including allergy. Here, we examined the role played by IECs in the effector phase of allergic responses following ingestion of allergen. The data summarized herein show that the canonical NF-κB signaling in IECs has no significant effect on allergic sensitization and the development of allergen-specific IgE responses. However, it regulates eotaxin production by IECs and thus, the recruitment of eosophils in the gut during the effector phase of allergic responses and the severity of clinical manifestations of disease.

In response to microbial stimuli or parasite infection, IECs produce cytokines, such as TLSP, which trigger a cascade of events leading to production of IgE. Recent evidence suggests that intrinsic signaling pathways in these cells could regulate production of other immunoglobulin isotypes. In this regard, our previous studies have shown that impaired canonical NF-κB signaling in IECs creates a cytokine microenvironment that facilitates production of IgA, and that oral sensitization of IKKβ^ΔIEC^ mice results in high levels of allergen-specific IgA in the bloodstream (13). The fact that clinical signs of allergy following nasal allergen challenge were less severe in orally sensitized IKKβ^ΔIEC^ mice and that this protection correlated with the levels of allergen-specific IgA in the secretions of the airways demonstrated the following two important points. First, it indicates that IECs can regulate the effector phase of allergic response at a distant mucosal site. It also supports the notion that the presence of allergen-specific SIgA at a mucosal site of allergen challenge can affect the effector phase of allergic response and limit the severity of disease. Here, we showed that orally sensitized IKKβ^ΔIEC^ mice are protected from the development of severe signs of allergy *via* mechanisms independent of IgA. As depicted in Figure 2, the titers of allergen-specific SIgA measured in fecal extracts were not higher than those seen in control wild-type mice. This finding confirms that allergen-specific serum and SIgA are differentially regulated. It also suggests that the higher levels of total IgA in fecal extracts of IKKβ^ΔIEC^ mice could result from increase secretion of IgA by peritoneal B1 cells in response to the inflammatory microenvironment.

Both the p65-mediated canonical and the p52-mediated non-canonical NF-κB pathways of neutrophils were reported to positively correlate with allergic symptom scores in human patients (28). Our findings indicate that the sensitization phase and IgE responses were not impaired by activation of p52 NF-κB in the absence of canonical NF-κB pathways in IECs and this was consistent with the fact that WT and IKKβ^ΔIEC^ mice displayed similar levels of *Tslp* and *Il-25* mRNA in the gut after oral allergen challenge. A previous report showed that IKKβ^ΔIEC^ mice are unable to produce TSLP and mount the mucosal Th2 responses required to eradicate infection with the gut-dwelling parasite Trichuris (29). Our data are consistent with the previous report that CT induced TSLP production by IECs (10) and demonstrate that TSLP can be induced even in the absence canonical NFκB signaling in IECs.

While the sensitization phase was not affected in our studies, a major finding from this work is the evidence that IECs can reduce allergic responses in the gut by limiting the recruitment of eosinophils. The Th2 cytokine IL-5 is crucial for the expansion and survival of eosinophils (30), but these cells are recruited to different sites by the chemokine CCL11 (eotaxin-1). Parenteral injection of cytokine is often the preferred route of delivery to address their regulatory effects of cytokines. However, this approach leads to high amounts of cytokines being present in the bloodstream and thus, may not allow to assess host responses to cytokines produced in mucosal tissues. We and other have previously shown that orally delivered cytokines can retain their biological activities (22–26). More importantly, we previously showed that even in the absence of significant levels of IL-12 in serum after oral administration, this treatment could promote IgG2a/c and suppress IgE antibody responses in mice orally immunized with CT as adjuvant (27). In the present study, oral administration of CCL11 helped established that the impaired/reduced production of this chemokine in the gut was the leading factor for reduced allergic responses despite high levels of allergen-specific IgE in the bloodstream.

Mast cells and basophils are major contributors to anaphylaxis. Eosinophils are other effector cells for allergic responses. Allergic responses from mast cell and basophil are IgE-dependent due to their expression of high-affinity IgE receptor (FcεRI) and develop during the early stage of exposure to allergen. Response from eosinophils develops slower and is more steady than that of those cells. Our data shows no difference in IgE level (Figure 1D), but differences in allergic behavior between WT and IKKβ-deficient mice. Thus, high clinical behavior were measured after 30 min in WT mice, while IKKβ-deficient mice only showed mild signs during the observation period. The gut microbiota is believed to play a significant role in the regulation of allergic responses and the presence of butyrate-producing bacteria is often associated with protection again the development of allergic responses (18–21). Our data show that the dysbiosis in IKKβ^ΔIEC^ mice was associated with reduced butyrate levels and thus suggest that lack of allergic symptoms in these mice was not due a protective effect of the microbiota or their products.

Intestinal epithelial cells were shown to produce CCL11 (31), and we now report that this process is regulated by the canonical NF-κB pathway in IECs. Our finding is consistent with the presence of an NF-κB binding site on the CCL11 promoter (32), and the previous report that inflammatory monocytes (F4/80^+^CD11b^+^Ly6^hi^) of mice lacking the canonical NF-κB in myeloid cells (RelA/p65^Δmye^ mice) have reduced expression of CCL11 (33). It is worth indicating that in a model of cutaneous and lung melanoma tumors, macrophages lacking IKKβ were reported to have enhanced production of CCL11 (34). Thus, while collectively these studies demonstrate a link between the canonical NF-κB pathway and CCL11, the suppressive or stimulatory effect of this signaling pathway on the production of this chemokine may differ depending of the cells and stimuli involved. Another point to consider is whether or not the impaired recruitment of eosinophils in the gut of IKKβ^ΔIEC^ mice has implication on other immune responses beside the lower magnitude of allergic responses. In this regard, eosinophils were recently shown to promote immunoglobulin class switch recombination and production of IgA by providing active TGFβ (35). Thus, the lower levels of allergen-specific mucosal IgA in the gut of IKKβ^ΔIEC^ mice could be related to the impaired production of CCL11 by IECs and subsequent reduced number of eosinophils in gut tissues.

Intestinal epithelial cells form the barrier that regulates the entry of exogenous pathogens and allergens. They have been more recently recognized as important members of the network of innate immune cells, which regulate the signature of the adaptive immune response through cytokines and chemokines they produce. Here, we show a new role of IECs in the regulation of the effector phase of allergic responses in the GI tract through their control of eotaxin production. This new knowledge sets the stage for future anti-allergy therapies targeting IKKβ signaling in IECs or oral delivery of anti-CCL11.

## Materials and Methods

### Mice

Control wild-type C57BL/6 mice were obtained from the Jackson Laboratory (Bar Harbor, ME, USA). C57BL/6 mice in which IKKβ-dependent NF-κB signaling was selectively eliminated in the IECs (IKKβ^ΔIEC^) were generated as previously described (36, 37). All animals were maintained under specific pathogen free conditions at The Ohio State University (OSU) animal care facility. Studies were approved by the Institutional Animal Care and Use Committee (IACUC, protocol #2008A02l0) and performed on mice aged 10–12 weeks, in accordance with NIH and OSU IACUC guidelines.

### Oral Sensitization and GI Antigen Challenge

Oral allergic sensitization and oral allergen challenge were performed as depicted in Figure 1A. For oral sensitization, mice were given 1 mg of OVA (grade V, Sigma-Aldrich, St. Louis, MO, USA) and 15 µg of CT (List Laboratories, Campbell, CA, USA) in 250 µl of phosphate-buffered saline (PBS), by intragastric gavage. Antigen challenge was performed eight times starting on day 14 by intragastric gavage of 50 mg of OVA (grade II, Sigma-Aldrich) in 500 µl of PBS.

### Assessment of Antigen-Specific IgG and IgA Ab Responses and Total IgA Levels

Total and OVA-specific Ab responses were measured in sera and fecal material extracts by enzyme-linked immunosorbent assay (ELISA) as previously described (13, 38). Briefly, microtiter plates were coated with OVA (1 mg/ml). For detection of OVA-specific IgG and IgA Abs, serial dilutions of serum or fecal material extract were added to the plates and the binding antibodies were detected with HRP-conjugated anti-mouse γ- or α-heavy chain-specific antisera (Southern Biotech Associates Inc., Birmingham, AL, USA). Biotin-conjugated rat anti-mouse IgG1, IgG2a/c, IgG2b, or IgG3 monoclonal Abs and HRP-conjugated streptavidin (BD Bioscience, San Jose, CA, ISA) were used to measure IgG subclass responses. The Ab titers were determined as the last dilutions of samples that with an absorbance of >0.1 above that of control samples from naïve mice.

Total IgA levels were determined by ELISA using extrapolation against IgA standards (38). Freshly emitted fecal pellets from IKKβ^ΔIEC^ and control mice were normalized by dissolution of 0.1 g feces in 1 ml of PBS, and the levels of IgA in fecal material extracts were further normalized by the amounts of total protein in each sample.

### Total and Antigen-Specific IgE Abs

Total IgE Ab levels were determined by a BD OptEIA™ Set Mouse IgE (BD PharMingen) according to instructions from the manufacturer. To prevent interference of IgG in the assay, serial dilutions of immune plasma were first depleted of IgG by overnight incubation in Reacti-Bind™ Protein G Coated Plates (Pierce, Rockford, IL, USA). In order to detect antigen-specific IgE, the microtiter plates were coated with OVA (1 mg/ml). Serial dilutions of IgG-depleted plasma were then added and IgE were detected with a biotinylated anti-mouse IgE Ab (BD Biosciences). The IgE titers were determined as the last dilution of samples that achieved an absorbance of >0.1 above that of control samples.

### Assessment of Surface Body Temperature

Body temperature was assessed by measuring surface body temperature with the aid of infrared thermometers [Heat spy infrared thermal imaging camera (Wahl, Culver City, CA, USA)].

### Clinical Signs of Allergy

Clinical behavior of allergy were evaluated for 30 min after oral allergen challenge by two independent researchers and assessed according to previously established guidelines (39). Briefly, the behavior associated with the following 0–5 scores are: 0 = no signs; 1 = scratching and rubbing around nose and head; 2 = reduced activity, self-isolation, and/or decreased activity with increased respiratory rate; 3 = motionless period lasting for more than 1 min; 4 = no activity against stimuli and/or convulsion; and 5 = death.

### Collection of Bronchoalveolar Lavage Fluids (BALF)

Bronchoalveolar lavage fluids were collected *via* cannulation of exposed trachea, by infusion of 1 ml of sterile PBS through a 22-Gauge catheter into the lungs, followed by aspiration of the fluid into a syringe. BALF samples were centrifuged (5 min at 1,500 rpm) and supernatants were collected and stored at −80°C and analyzed as previously reported (13).

### Assessment of Histamine Levels

Serum histamine levels were assessed in samples collected 2 h after the last oral allergen challenge using a histamine ELISA kit (My Biosource, San Diego, CA, USA) and according to manufacturer’s instruction.

### Measure of Cytokines and Chemokines

The concentration of cytokines and chemokines in biological fluids (i.e., serum or BALF) and culture supernatants were evaluated by multiplex assay using the Mouse Cytokine 23-plex Panel (Bio-Rad, Hercules, CA, USA) and according to manufacturer’s instruction.

### Histology

Immunohistochemistry was performed on thin (5 µm) sections of formalin-fixed and paraffin-embedded tissues. Intestines were extensively washed with cold PBS before fixation. Tissue sections were stained with Periodic Acid-Shiff. For immunohistochemistry, tissues were stained with the following antibodies (Santa Cruz Biotechnology, Dallas, TX, USA): anti-p100/p52 (dilution 1:100); anti-Siglec-F (dilution 1:100), and anti-CCL11 (Abcam, Cambridge, MA, USA, dilution 1:50) and secondary fluorescent Abs. Nuclei were counterstained with DAPI.

### Intestinal Epithelial Cell Line

The human intestinal epithelial cell line HT-29 and the murine intestinal epithelial cell line CMT93 were was cultured in DMEM (Gibco, Rockville, MD, USA) containing 10% fetal calf serum. To address the effect of NF-κB inhibition on steady-state or cytokine responses to CT, HT-29 cells were incubated in the presence of the specific NF-κB inhibitor sulfasalazine (Sigma-Adrich, St. Louis, MO, USA).

### Real-Time PCR

Tissues were collected, snap frozen, and reduced to powder before adding TRIzol (Invitrogen, Carlsbad, CA, USA). The cDNA was synthesized by using Superscript III (Invitrogen) and real-time PCR was performed as previously described (40) with the aid of primers listed in Table 1. The data were expressed as relative mRNA expression = 2^−ΔΔCt^ where ΔCt = Ct_unknown_ − Ct_HKG_, and normalized against two house-keeping genes: β-actin and hypoxanthine-guanine phosphoribosyl-transferase. The list and sequence of primers are provided in Table 1.

**Table 1:** List of primers used for real-time RT-PCR.

Species	Name		Sequence	Size
Mouse	*Hprt1*	F	GAG GAG TCC TGT TGA TGT TGC CAG	173
		R	GGC TGG CCT ATA GGC TCA TAG TGC	
	
	β*-actin*	F	GCG CAA GTA CTC TGT GTG GA	162
		R	GAA AGG GTG TAA AAC GCA GC	
	
	*Ccl11*	F	TCC TTC ATG ACC TTG TGC AG	162
		R	GGA ATA GAA GCG CTG TGG AG	
	
	*Ccl24*	F	AGC CTT CTA AAG GGG CCA AG	140
		R	CCC CAA AGC AGC CTG GTA AA	
	
	*Il-25*	F	GGA TGG CCC CCT CAA CA	66
		R	CGA TTC AAG TCC CTG TCC AAC T	
	
	*Tslp*	F	GCT TGT CTC CTG AAA ATC GAG TAT	83
		R	CTC CGG GCA AAT GTT TTG TC	
	
	*Il-5*	F	CAG TGT GTA GCC AAG GGT GAC	166
		R	TGA AGT TAG ATA GGA GCA GGA AGC	
	
	*Epx*	F	TCA GAT TGT GCG CTT CCC CAG C	164
		R	GGG CAC AGG TCT TCT CAC AGT CCA	
	
Human	β*-ACTIN*	F	TGG GCA TGG GTC AGA AGG AT	84
		R	GCT CGA TGG GGT ACT TCA GG	
	
	*CCL11*	F	GGG CCA GCT TCT GTC CCA AC	225
		R	TTA TGG CTT TGG AGT TGG AGA TTT	
	
	*IL-1*β	F	AAA TAC CTG TGG CCT TGG GC	101
		R	TTT GGG ATC TAC ACT CTC CAG CT	
	
	*TNF*α	F	CCC AGG GAC CTC TCT CTA ATC A	104
		R	GCT TGA GGG TTT GCT ACA ACA TG	

### Epithelial Cell Organoid Cultures

Intestinal organoids of wild-type C57BL/6 and IKKβ^ΔIEC^ mice were generated by culture of crypts of small intestine in Intesti Cult™ growth media (STEMCELL technologies, Cambridge, MA, USA) as previously reported (41, 42). Briefly, intestinal crypts were isolated from small intestine of wild-type or IKKβ^ΔIEC^ mice by incubation with 2 mM of EDTA for 30 min at 4°C, mixed with 50 µl of Matrigel (BD Bioscience) and placed in pre-warmed 24-well culture plates. The numbers of organoids were maintained around 200 per well.

### Oral Treatment With Recombinant CCL11

The oral treatment with a recombinant CCL11 (R&D Systems, Minneapolis, MN, USA) was performed as previously described with modifications (27). Briefly, groups of sensitized mice were deprived of food for 2 h, followed by intragastric administration of an isotonic bicarbonate solution (8 parts HBSS and 2 parts 7.5% sodium bicarbonate) to neutralize stomach acidity. Individual mice were gavaged with a 0.1 ml PBS solution (pH 7.2) containing 1 µg of recombinant CCL11 and 30 min later, were gavaged with 50 mg of OVA in 0.5 ml of PBS.

### Analysis of Gut Microbiota

Freshly emitted fecal pellets were collected for all studies. Briefly, freshly emitted fecal pellets samples were collected, normalized weight, snap frozen, and stored at −80°C. Samples were collected twice a day from each individual mouse and pooled to minimize potential daily variation of the microbiota. The bacterial tag-encoded FLX amplicon pyrosequencing (Roche, Branford, CT, USA) was used for identification of primary populations of microbes in fecal pellets as previously described (13). Briefly, Bacterial DNA was extracted by conventional methods (Qiagen, Valencia, CA, USA), and 16S rRNA genes were amplified with the modified 16S Eubacterial primers 27F, 5-GAG TTT GAT CNT GGC TCA G-3′ and 519R, 5-GTN TTA CNG CGG CKG CTG-3′ for amplifying the 500 bp region of 16S rRNA genes. The primer sets used for FLX-Titanium amplicon pyrosequencing were designed with adding linker A and 8 base pair barcode sequence at the 5′ end of forward primers as follow: 27 F-A, 5-CCA TCT CAT CCC TGC GTG TCT CCG ACT CAG-barcode-GAG TTT GAT CNT GGC TCA G-3′. The biotin and linker B sequence at the 5′ end of reverse primer 519R-B: 5-Biotin-CCT ATC CCC TGT GTG CCT TGG CAG TCT CAG GTN TTA CNG CGG CKG CTG-3′. HotStarTaq Plus Master Mix Kit (QIAGEN, CA, USA) was used for PCR under the following conditions: 95°C for 5 min followed by 35 cycles of 95°C for 30 s, 54°C for 40 s, and 72°C for 1 min, a final elongation step at 72°C for 10 min was also included. The PCR products were cleaned by using Diffinity Rapid Tip (Diffinity Genomics, Inc., West Henrietta, NY, USA), and the small fragments were removed by using Agencourt Ampure Beads (Beckman Coulter, CA, USA). Bacterial tag-encoded FLX-Titanium amplicon pyrosequencing (bTEFAP) was performed as described previously (43). In preparation for FLX-Titanium sequencing (Roche, Nutley, NJ, USA), DNA fragment sizes and concentration were accurately measured using DNA chips under a Bio-Rad Experion Automated Electrophoresis Station (Bio-Rad Laboratories, CA, USA) and a TBS-380 Fluorometer (Turner Biosystems, CA, USA). A sample of double-stranded DNA, 9.6 million molecules/ml, with an average size of 625 bp were combined with 9.6 million DNA capture beads, and then amplified by emulsion PCR. After bead recovery and bead enrichment, the bead attached DNAs were denatured with NaOH, and sequencing primers (Roche) were annealed. A four-region 454 sequencing run was performed on a GS PicoTiterPlate (PTP) using the Genome Sequencer FLX System (Roche). Forty tags were used on each quarter region of the PTP. All FLX procedures were performed using Genome Sequencer FLX System manufacturer’s instructions (Roche). After denoising (USEARCH application) and chimera removal (UCHIIME in *de novo* mode), the sequences were clustered into operational taxonomic units clusters with 96.5% identity (3.5% divergence) using USEARCH and the seed sequence put into a FASTA formatted sequence file. The FASTA files were then queried against a database of high quality sequences derived from NCBI using a distributed.NET algorithm that utilizes BLASTN + (KrakenBLAST www.krakenblast.com). The Bray–Curtis index (44) and Principal component analyses (PCA) was used to summarize the relationship between microbial communities in the wild-type and IKKβ^ΔIEC^ mice. LDA scores were analyzed using the Galaxy software (https://huttenhower.sph.harvard.edu/galaxy/) and the threshold on the logarithmic scale for discriminative features was set at >2.0 (45).

### Metabolomics

Metabolite profiles of small intestinal and cecal samples were analyzed by proton nuclear magnetic resonance (NMR). Intestinal scrapings were resuspended in phosphate buffer (4.3 mM Na_2_HPO_4_⋅7H_2_O, 1.5 mM KH_2_PO_4_, 2.7 mM KCl), homogenized, and filtered as described (46). A 70 µl aliquot of prepared extract was mixed with 19 µl of 9 mM trimethylsilylpropionic-2,2,3,3-d_4_ acid (TSP) in D_2_O. The sample was transferred into a micro coaxial NMR tube insert (60 µl volume, 2.02 mm OD, Wilmad Lab Glass, Co.), which was then placed inside a 5 mm NMR tube. Proton (^1^H) NMR spectra were acquired at 25°C using a Varian INOVA operating at 600 MHz (14.1 T) as we described previously (47), with an acquisition time of 4 s, interpulse delay of 6.55 s, and 1,680 transients (3 h of signal averaging). Spectra were pre-processed using Varian software and were baseline corrected using the Whittaker Smoother algorithm. For multivariate data analyses, spectra were binned to reduce the dimensionality and mitigate peak misalignment (46). A dynamic programming-based adaptive binning technique was employed (48) using a minimum and maximum distance between peaks in a single bin of 0.001 and 0.04 ppm, respectively. Quantification of specific metabolite resonances was accomplished using an interactive spectral deconvolution algorithm in Matlab (48). All metabolite peak intensities were corrected for equivalent number of protons and normalized relative to the TSP signal intensity. Peaks were assigned to specific metabolites following a previously described procedure (46). To assess metabolome similarity among samples, principal components analysis (PCA) was run on the binned dataset; the intra-group variance was adjusted by Mahalanobis scaling as we did previously (47). Separation of sample groups in PCA ordination space was tested based on the permutation analysis of the Davies–Bouldin index measure (49).

### Statistical Analysis

Results are expressed as the mean ± 1 SD. Statistical significance was determined by one-way ANOVA, followed by Tukey *post hoc* test. All statistical analyses were performed with the StataSE 12.0 software (StataCorp LLC, College Station, TX, USA) and Prism 7 software (Graphpad Software, La Jolla, CA, USA).

## Ethics Statement

Studies were approved by the Institutional Animal Care and Use Committee (IACUC, protocol #2008A02l0) and performed on mice aged 10–12 weeks, in accordance with NIH and OSU IACUC guidelines.

## Author Contributions

Designed studies and wrote the manuscript: EK, EC-B, and PB; conducted studies: EK, ML, GF, JR, TM, NR; analyzed data: EK, NR, OP, AS, EC-B, and PB; edited the manuscript: EK, AS, OP, EC-B, and PB.

## Conflict of Interest Statement

The authors declare that the research was conducted in the absence of any commercial or financial relationships that could be construed as a potential conflict of interest.
